# Comparison of instrumented and stand-alone lateral lumbar interbody fusion for lumbar degenerative disease: a systematic review and meta-analysis

**DOI:** 10.1186/s12891-024-07214-6

**Published:** 2024-02-03

**Authors:** Lianghai Jiang, Lantao Liu, Liang Dong, Zhengwei Xu, Xiaobo Zhang, Lixiong Qian

**Affiliations:** 1 Department of Spine Surgery, Qingdao Municipal Hospital, Qingdao, People’s Republic of China; 2 Department of Orthopedic, Honghui Hospital, Xi’an Jiaotong University College of Medicine, Xi’an, People’s Republic of China

**Keywords:** Degenerative disease, Fusion, Lumbar, Stand-alone

## Abstract

**Background:**

Both instrumented and stand-alone lateral lumbar interbody fusion (LLIF) have been widely used to treat lumbar degenerative disease. However, it remains controversial as whether posterior internal fixation is required when LLIF is performed. This meta-analysis aims to compare the radiographic and clinical results between instrumented and stand-alone LLIF.

**Methods:**

PubMed, EMBASE and Cochrane Collaboration Library up to March 2023 were searched for studies that compared instrumented and stand-alone LLIF in the treatment of lumbar degenerative disease. The following outcomes were extracted for comparison: interbody fusion rate, cage subsidence rate, reoperation rate, restoration of disc height, segmental lordosis, lumbar lordosis, visual analog scale (VAS) scores of low-back and leg pain and Oswestry Disability Index (ODI) scores.

**Results:**

13 studies involving 1063 patients were included. The pooled results showed that instrumented LLIF had higher fusion rate (OR 2.09; 95% CI 1.16–3.75; *P* = 0.01), lower cage subsidence (OR 0.50; 95% CI 0.37–0.68; *P* < 0.001) and reoperation rate (OR 0.28; 95% CI 0.10–0.79; *P* = 0.02), and more restoration of disc height (MD 0.85; 95% CI 0.18–1.53; *P* = 0.01) than stand-alone LLIF. The ODI and VAS scores were similar between instrumented and stand-alone LLIF at the last follow-up.

**Conclusions:**

Based on this meta-analysis, instrumented LLIF is associated with higher rate of fusion, lower rate of cage subsidence and reoperation, and more restoration of disc height than stand-alone LLIF. For patients with high risk factors of cage subsidence, instrumented LLIF should be applied to reduce postoperative complications.

**Supplementary Information:**

The online version contains supplementary material available at 10.1186/s12891-024-07214-6.

## Introduction

Lumbar degenerative disease is a common condition treated by spine surgeons, which can induce dysfunction and decrease of quality of life. Lateral lumbar interbody fusion (LLIF), including extreme/direct lateral interbody fusion [1, 2], and oblique lateral interbody fusion (OLIF) [3], has been used to treat lumbar degenerative disease. LLIF is a minimally invasive technique with satisfactory result in indirect decompression of spinal canal and foramina.

Both instrumented and stand-alone LLIF have been widely used and proved effective in clinical work. However, it remains controversial as whether posterior internal fixation is required when LLIF is performed [4]. Some reports showed that stand-alone LLIF could achieve equivalent clinical and radiological results like instrumented LLIF [5]. And stand-alone LLIF is associated with short operation time, small trauma, and much more cost-effective [6, 7]. On the other hand, some authors argue that instrumented LLIF has lower rate of postoperative complications [8] including cage subsidence, nonunion, and reoperation. There is still a lack of evidence-based medicine to prove the clinical results between instrumented and stand-alone LLIF. Therefore, we performed a systematic review and meta-analysis to compare the efficacy between instrumented and stand-alone LLIF.

## Materials and methods

### Inclusion criteria

The inclusion criteria of this meta-analysis: (1) target population: patients with lumbar degenerative disease including disc herniation, stenosis, spondylolisthesis and so on; (2) intervention: LLIF with posterior instrumentation (instrumented LLIF) versus stand-alone LLIF. Only studies comparing these two techniques were included; (3) methodological criteria: prospective or retrospective trials. Reviews, case reports and biomechanical analysis were excluded. Studies that could not provide adequate information on the mean or odds ratio were excluded.

### Search strategy

The PubMed, EMBASE and Cochrane Collaboration Library up to March 2023 were searched using the following terms: “lateral lumbar interbody fusion”, “extreme lateral interbody fusion”, “direct lateral interbody fusion”, “oblique lateral interbody fusion”, “stand alone”, “stand-alone”, “standalone”. Two authors (L.J. and Z.X.) screened the relevant studies independently.

### Quality assessment

Quality of the included studies was assessed independently by two authors (X.Z. and L.Q.). The Newcastle Ottawa Quality scale [9] was used to for the assessment of prospective or retrospective studies.

### Data extraction

Data extraction was performed by two authors (L.J. and L.L.) independently. General characteristics of the included studies were recorded: study design, year of publication, first author, sample size, and follow-up time. The clinical and radiographic outcomes were extracted from studies for comparison: interbody fusion rate, cage subsidence rate, reoperation rate, restoration of disc height, segmental lordosis, lumbar lordosis, visual analog scale (VAS) scores of low-back and leg pain and Oswestry Disability Index (ODI) scores.

### Statistical analysis

The abstracted data were analyzed using Review Manager version 5.3 (Cochrane Collaboration). Continuous data were presented in terms of mean difference (MD) and 95% confidence interval (CI); and dichotomous data were presented in terms of odds ratio (OR) and 95% CI. Statistical heterogeneity among the studies was checked using the χ2 test. *P* > 0.10 or I^2^ < 50% indicated that there was no significant heterogeneity, and the fixed-effects model was used. Otherwise, *P* < 0.10 or I^2^ > 50% indicated significant heterogeneity. The random-effects model was used when the source of heterogeneity could not be found.

## Results

### Literature search

**Fig. 1:**
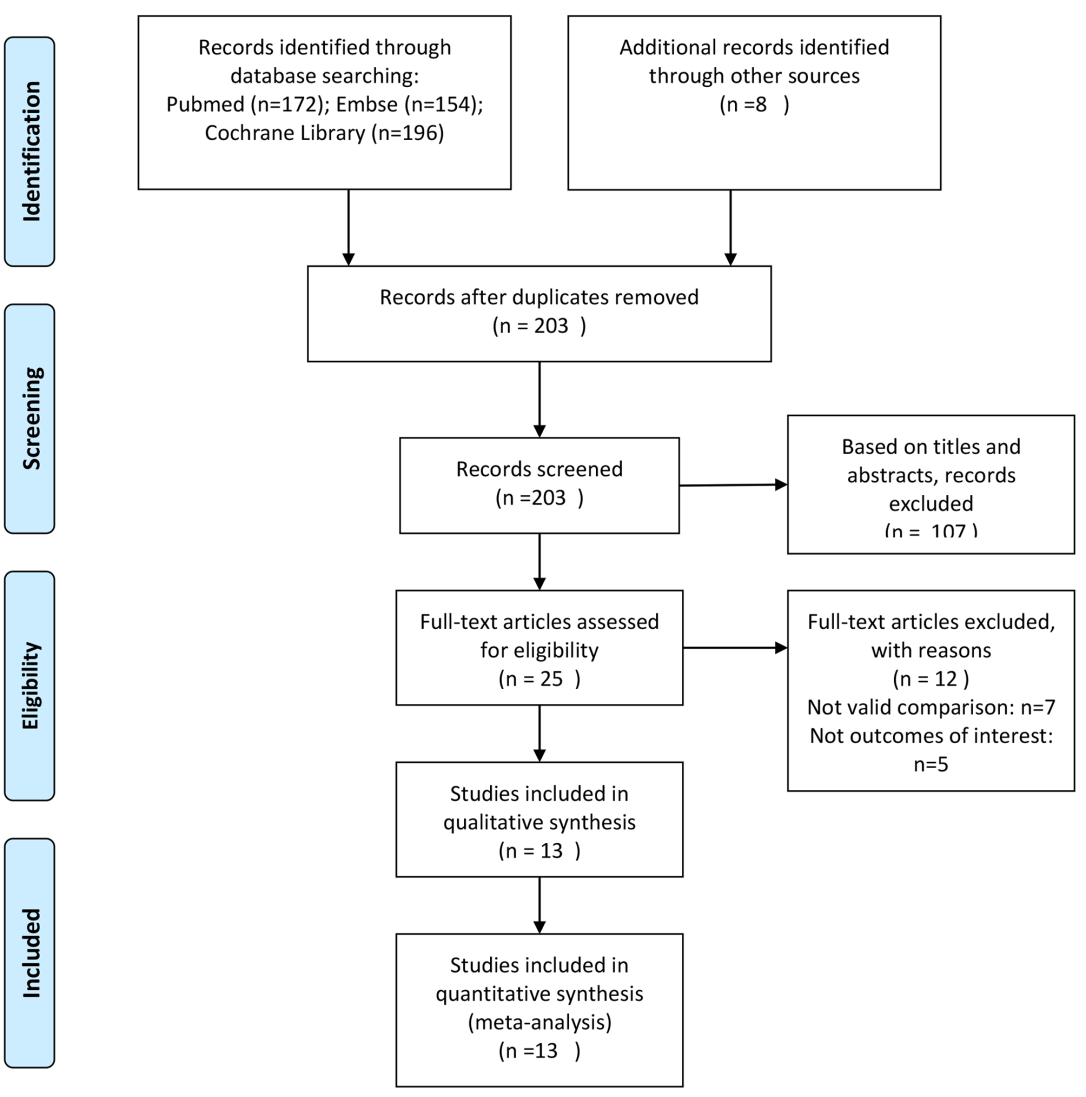
Flow diagram depicting the literature search and selection process

Based on the inclusion criteria, 203 articles were found in the database. 190 studies were removed after reviewing the titles, abstracts or full text. Finally, 13 studies [5, 10–21] involving 1063 patients (instrumented group 581, stand-alone group 482) were included in the meta-analysis. A detailed flowchart of steps of literature search is shown in Fig. 1.

### Quality of the Individual studies

The 13 studies included 3 prospective studies [11, 17, 19] and 10 retrospective studies [5, 10, 12–16, 18, 20, 21]. Quality of the included studies was evaluated according to the Newcastle-Ottawa Scale. Of the 13 studies, ten were high-quality with scores 8–9, and three were moderate with a score of 7 (Table 1). Baseline characteristics of included studies were shown in Table 2.

**Table 1:** Quality assessment of the studies included according to newcastle-ottawa scale

Author	Year	Selection	Comparability	Exposure	Total Score
Cheng	2021	3	2	3	8
Wu	2021	3	2	3	8
Li	2021	3	2	3	8
Jones	2021	4	2	3	9
Cai	2021	3	2	3	8
He	2020	3	2	3	8
Chen	2019	3	2	3	8
Parker	2017	4	2	3	9
Malham	2017	3	1	3	7
Aichmair	2017	3	2	3	8
Malham	2012	3	2	3	8
Kim	2012	3	1	3	7
Sharma	2011	3	1	3	7

**Table 2:** Baseline characteristics of studies included in this meta-analysis

Author	Year	Design	Treatment (LLIF)	Sample size	Age (years)	Sex (Male/Female)	Follow-up (months)
Cheng	2021	Retrospective	Instrumented Stand-alone	15 48	67.0 ± 10.0	NA	23.2 ± 11.5
Wu	2021	Retrospective	Instrumented Stand-alone	25 36	65.1 ± 9.5	30/31	12
Li	2021	Prospective	Instrumented Stand-alone	41 54	57.9 ± 8.2/60.3 ± 6.2	20/21 19/35	17.1 ± 3.5 16.3 ± 4.0
Jones	2021	Retrospective	Instrumented Stand-alone	239 108	61.7 ± 11.1	174/173	12
Cai	2021	Retrospective	Instrumented Stand-alone	25 41	62.16 ± 8.65/59.46 ± 8.46	12/13 22/19	≥ 6
He	2020	Retrospective	Instrumented Stand-alone	41 32	61.0 ± 9.3/59.8 ± 13.7	11/30 10/22	24
Chen	2019	Retrospective	Instrumented Stand-alone	26 27	61.5 ± 10.9/60.2 ± 11.3	7/19 15/12	24
Parker	2017	Retrospective	Instrumented Stand-alone	78 54	31–86	45/87	24
Malham	2017	Prospective	Instrumented Stand-alone	19 21	61.8 ± 10.3/65.2 ± 12.1	5/14 7/14	12
Aichmair	2017	Retrospective	Instrumented Stand-alone	21 31	60.9 ± 10.6/62.5 ± 12.1	35/17	16.1 ± 9.8
Malham	2012	Prospective	Instrumented Stand-alone	14 16	62.7 ± 10.5	10/20	12
Kim	2012	Retrospective	Instrumented Stand-alone	4 4	64.0 ± 9.6/67.5 ± 8.3	1/3 1/3	2–8
Sharma	2011	Retrospective	Instrumented Stand-alone	33 10	63.9 ± 10.2	16/27	12

### Radiographic outcomes

#### Fusion rate

**Fig. 2:**
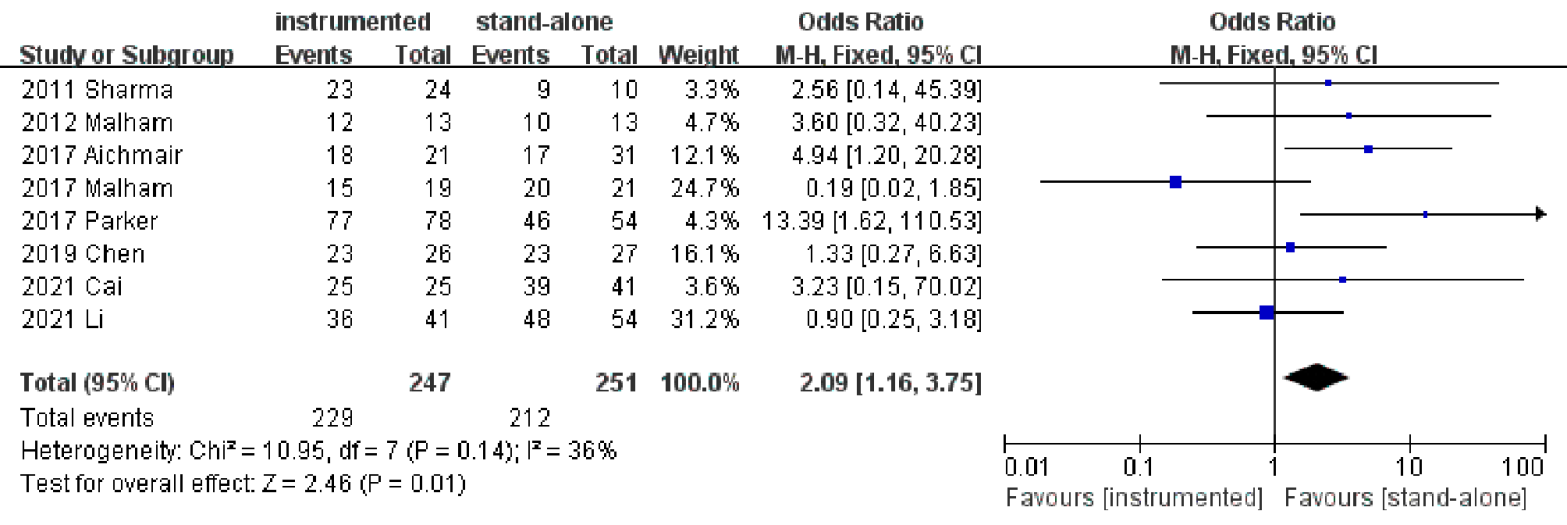
Forest plots of fusion rate in instrumented and stand-alone groups

Eight studies [11, 14–19, 21] presented the information of fusion rate at the last follow-up. Pooled results revealed a significantly higher fusion rate in the instrumented group than the stand-alone group (OR 2.09; 95% CI 1.16–3.75; *P* = 0.01; heterogeneity: *P* = 0.14, I^2^ = 36%, fixed-effects model, Fig. 2).

#### Cage subsidence

**Fig. 3:**
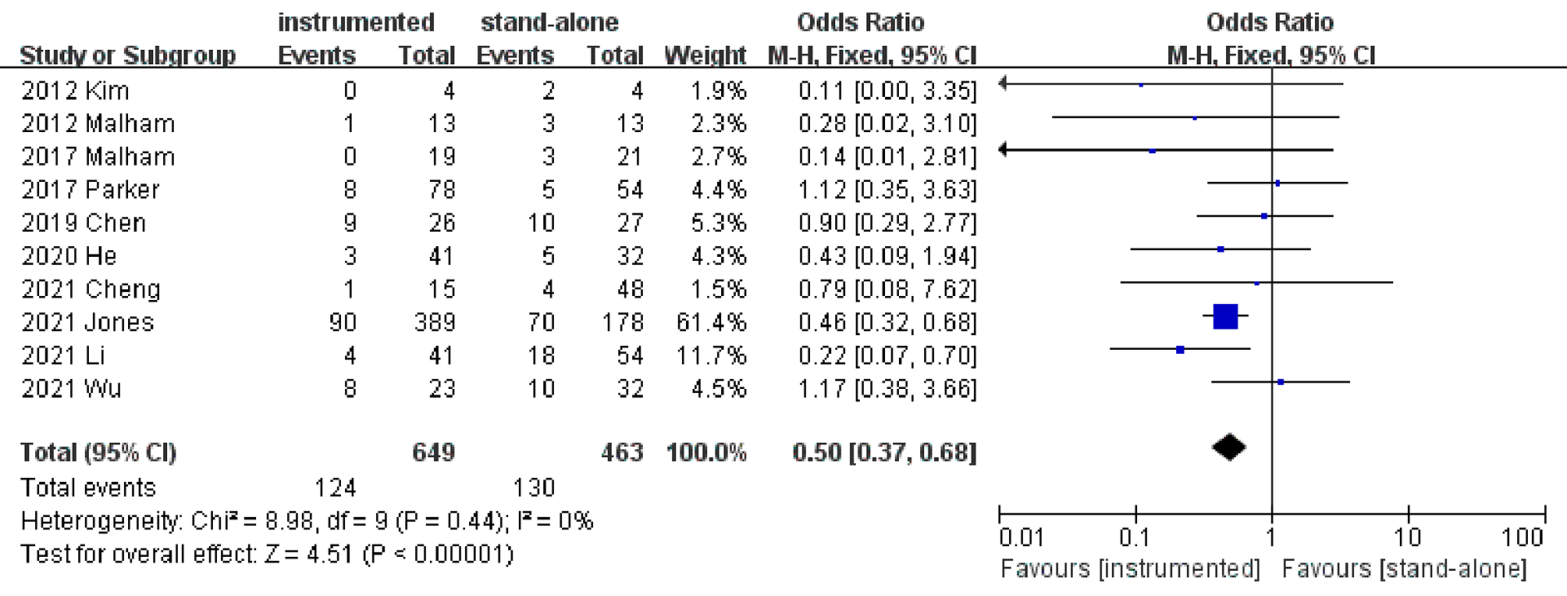
Forest plots of cage subsidence rate in instrumented and stand-alone groups

**Fig. 4:**
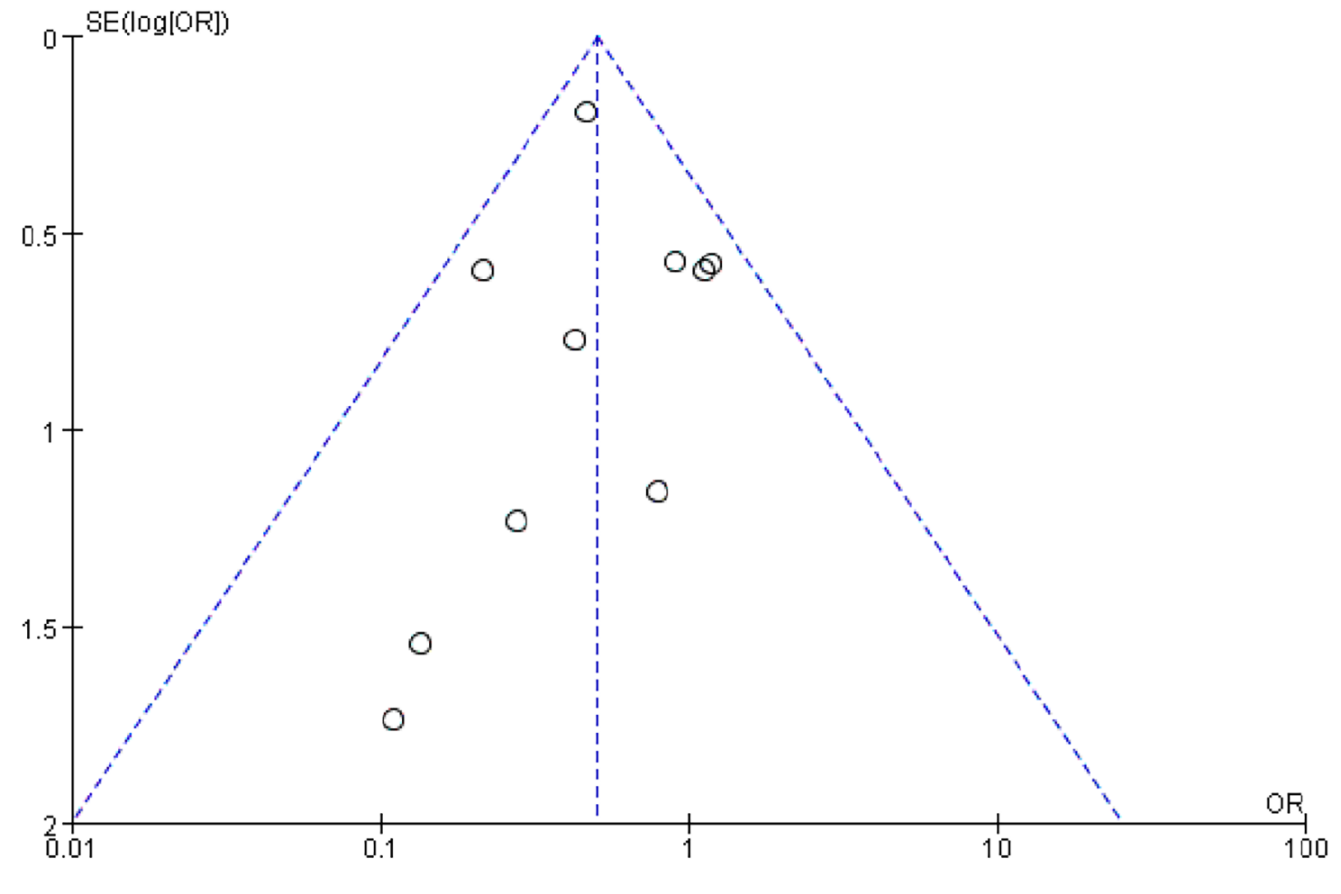
Funnel plot for cage subsidence rate to assess publication bias among included studies

Ten studies [5, 10–13, 15–17, 19, 20] presented the cage subsidence rate at the last follow-up. Pooled results revealed a significantly lower cage subsidence rate in the instrumented group than the stand-alone group (OR 0.50; 95% CI 0.37–0.68; *P* < 0.001; heterogeneity: *P* = 0.44, I^2^ = 0%, fixed-effects model, Fig. 3). Funnel plot for the cage subsidence rate was used to assess the publication bias. As the funnel plot appeared symmetrical, no significant publication bias was found in this meta-analysis (Fig. 4).

#### Reoperation rate

**Fig. 5:**
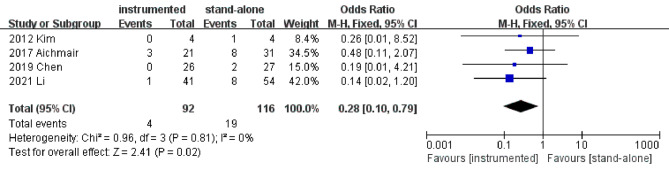
Forest plots of reoperation rate in instrumented and stand-alone groups

Four studies [11, 15, 18, 20] reported the data of reoperation rate at the last follow-up. According to the pooled results, the instrumented group had a significantly lower reoperation rate than the stand-alone group (OR 0.28; 95% CI 0.10–0.79; *P* = 0.02; heterogeneity: *P* = 0.81, I^2^ = 0%, fixed-effects model, Fig. 5).

#### Disc height

**Fig. 6:**
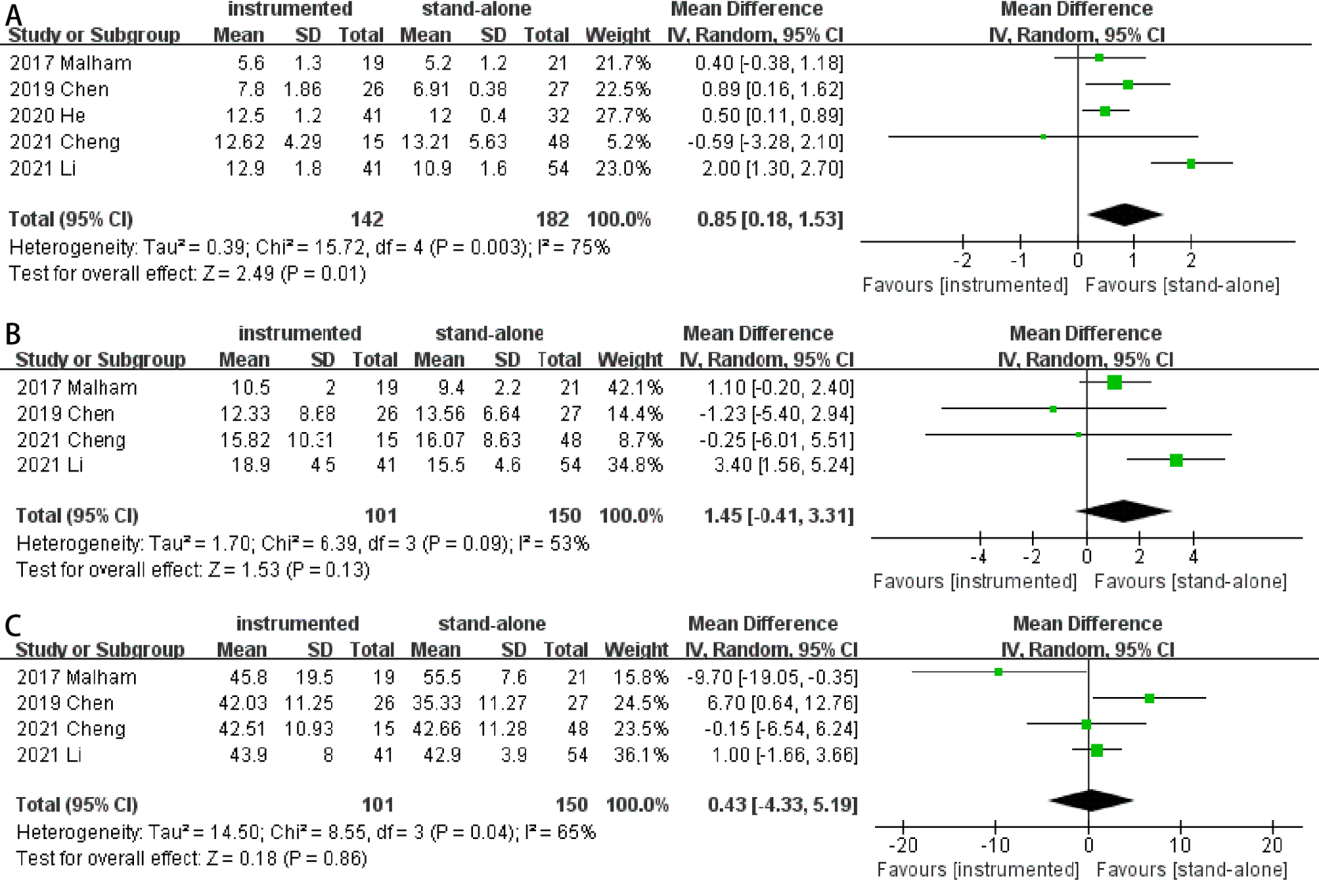
Forest plots of restoration of disc height (**A**), segmental lordosis (**B**), and lumbar lordosis (**C**) in instrumented and stand-alone groups

Five studies [5, 11, 13, 15, 17] reported the restoration of disc height. Preoperative disc height between the two groups were similar (MD -0.51; 95% CI -1.35- 0.33; *P* = 0.24; heterogeneity: *P* = 0.01, I^2^ = 70%, random-effects model). At the last follow-up, pooled results showed the instrumented group had significantly more restoration of disc height (MD 0.85; 95% CI 0.18–1.53; *P* = 0.01; heterogeneity: *P* = 0.003, I^2^ = 75%, random-effects model) (Fig. 6A).

#### Segmental and lumbar lordosis

Four studies [11, 13, 15, 17] reported the restoration of segmental and lumbar lordosis. Preoperative segmental lordosis (MD -0.83; 95% CI -1.86- 0.19; *P* = 0.11; heterogeneity: *P* = 0.71, I^2^ = 0%, fixed-effects model) and lumbar lordosis (MD 1.10; 95% CI -1.97- 4.17; *P* = 0.48; heterogeneity: *P* = 0.29, I^2^ = 20%, fixed-effects model) between the two groups were similar. At the last follow-up, no significant differences were found between instrumented and stand-alone groups in segmental lordosis (MD 1.45; 95% CI -0.41- 3.31; *P* = 0.13; heterogeneity: *P* = 0.09, I^2^ = 53%, random-effects model; Fig. 6B) or lumbar lordosis (MD 0.43; 95% CI -4.33- 5.19; *P* = 0.86; heterogeneity: *P* = 0.04, I^2^ = 65%, random-effects model Fig. 6C).

### Clinical outcomes

#### ODI score

**Fig. 7:**
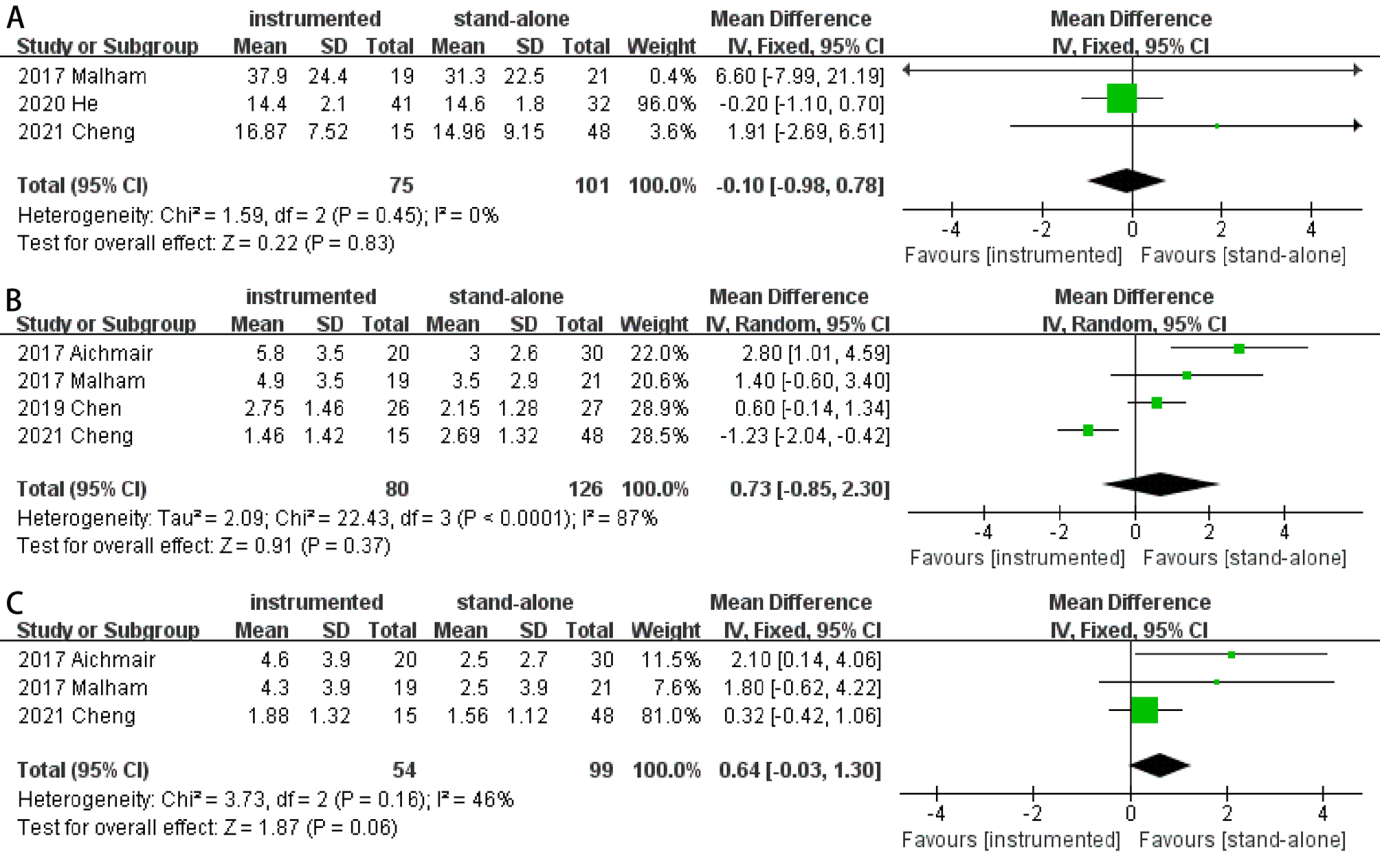
Forest plots of ODI score (**A**), VAS score for low back (**B**), and VAS score for leg (**C**) in instrumented and stand-alone groups

Based on three studies [5, 13, 17], preoperative ODI score between the two groups were similar (MD 0.72; 95% CI -0.58- 2.02; *P* = 0.28; heterogeneity: *P* = 0.79, I^2^ = 0%, fixed-effects model). At the last follow-up, pooled results showed there was no significant difference between two groups in ODI score (MD -0.10; 95% CI -0.98- 0.78; *P* = 0.83; heterogeneity: *P* = 0.45, I^2^ = 0%, fixed-effects model; Fig. 7A).

#### VAS score

A total of four studies [13, 15, 17, 18] reported the VAS scores of low-back and leg. There were no significant differences in preoperative VAS scores of low-back (MD 0.25; 95% CI -0.17- 0.67; *P* = 0.24; heterogeneity: *P* = 0.55, I^2^ = 0%, fixed-effects model) or leg (MD -0.29; 95% CI -1.01- 0.42; *P* = 0.42; heterogeneity: *P* = 0.59, I^2^ = 0%, fixed-effects model). At the last follow-up, pooled results showed no significant difference in VAS score of low-back (MD 0.73; 95% CI -0.85- 2.30; *P* = 0.37; heterogeneity: *P* < 0.001, I^2^ = 87%, random-effects model) or leg (MD 0.64; 95% CI -0.03- 1.30; *P* = 0.06; heterogeneity: *P* = 0.16, I^2^ = 46%, fixed-effects model) between two groups (Fig. 7BC).

## Discussion

For years, as a minimally invasive approach, LLIF is one of the most commonly used techniques [1, 22–25]. Both instrumented and stand-alone LLIF have been widely performed in clinic work [26–28]. Some studies noted that stand-alone method could be sufficient to achieve stabilization and fusion [17, 29–31]. However, other studies proved that stand-alone LLIF are associated with higher rate of nonunion and cage subsidence [11], which would impact the clinical outcome. Hence, we perform this meta-analysis to compare the efficacy between instrumented and stand-alone LLIF for lumbar degenerative disease.

Fusion is of great importance for patients who underwent LLIF. In the previous systematic review by Manzur et al., the instrumented LLIF group had a higher fusion rate than the stand-alone group (91.0% vs. 80.4%) [32]. Similarly, our meta-analysis showed the fusion rate was higher in the instrumented group than the stand-alone group (92.7% vs. 84.5%, *P* = 0.01). The higher fusion rate may be result from sufficient rigidity and limited range of motion provided by posterior fixation [33] .

Cage subsidence is one of the most common complications after LLIF [34–39], which is associated with factors like osteoporosis, endplate violation, and higher BMI. This meta-analysis showed the instrumented group had lower cage subsidence rate than the stand-alone group. This result can be explained by the fact that posterior instrumentation could improve the stability and distribute load across the endplate. Therefore, patients with high risk factors of cage subsidence are advised to take the instrumented LLIF. On the contrary, for patients without the risk factors of cage subsidence including osteoporosis, endplate violation, and higher BMI, stand-alone LLIF can be considered. This meta-analysis also showed the instrumented group had more restoration of disc height at the last follow-up, which is related to the lower cage subsidence rate.

With respect to clinical outcomes, previous review by Alvi et al. demonstrated comparable ODI and VAS scores between instrumented and stand-alone groups at the last follow-up [8], which was consistent with the results in our study. Though the stand-alone group had a higher rate of cage subsidence, most cases were low-grade subsidence and were mostly asymptomatic. However, it should be noted that only three studies were included in the comparison of ODI score, and significant heterogeneity was detected in the in the comparison of low-back VAS score. More high quality studies are needed for further evaluation. Clinical outcomes are also highly correlated with spinal alignment and spinopelvic parameters (pelvic index, pelvic tilt, sacral slope, sagittal vertical axis) [40], as well as spino-pelvic-femoral parameters such as femoral obliquity angle (FOA) and T1 pelvic angle (TPA). FOA > 10°and increased TPA were reported to be associated with worse clinical and functional outcomes [41]. Spinopelvic and spino-pelvic-femoral parameters should be considered in the future meta-analysis.

There are several limitations in this meta-analysis. First, there is no randomized controlled trial included in this study. Second, the number of patients included in the meta-analysis is relatively small. Third, patients included in this meta-analysis had different lumbar degenerative disease including degenerative disc disease, spondylolisthesis, adjacent segment disease, scoliosis and so on. All the lumbar degenerative disease were put together in this meta-analysis, which may lead to significant heterogeneity.

In summary, based on this meta-analysis, instrumented LLIF is associated with higher rate of fusion, lower rate of cage subsidence and reoperation, and more restoration of disc height than stand-alone LLIF. For patients with high risk factors of cage subsidence, instrumented LLIF should be applied to reduce postoperative complications.

### Electronic supplementary material

Below is the link to the electronic supplementary material.

Supplementary Material 1

## Data Availability

The data and materials contributing to this article may be made available upon request by sending an e-mail to the corresponding author.

## Abbreviations

LLIF: Lateral lumbar interbody fusion
OLIF: Oblique lateral interbody fusion
VAS: Visual analog scale
ODI: Oswestry Disability Index
CI: Confidence interval
OR: Odds ratio
FOA: Femoral obliquity angle
TPA: T1 pelvic angle
